# 2293: Passive intracranial EEG-based localization of the central sulcus during sleep

**DOI:** 10.1017/cts.2017.67

**Published:** 2018-05-10

**Authors:** Rafeed Alkawadri

**Affiliations:** Yale School of Medicine, Guilford, CT, USA

## Abstract

OBJECTIVES/SPECIFIC AIMS: To investigate the performance of a metric for passive localization of central sulcus. METHODS/STUDY POPULATION: We studied 7 patients with intractable epilepsy undergoing intra-cranial EEG (icEEG) monitoring at Yale, in whom central sulcus (CS) localization was obtained by standard methods. Our method takes advantage of inherent properties of the primary motor cortex (MC), which exhibits enhanced icEEG band-power and coherence across the CS. For each contact *x* we calculated the *z*-score of a composite power and synchrony value log_10_(px)x;cx, where px is sum of the root mean square of the icEEG in the high gamma band (80–115 Hz) for contact *x* over the 6–10 minutes of NREM sleep studied, and cx is the mean magnitude squared coherence in the same band using a 500-ms Hamming window between contact *x* and all other contacts. *z*-score values lower than threshold (th) were set to 0. Finally, we calculated a metric *m*=*z*/*d*, where *d* is the mean Euclidian distance of each contact from contacts with *z* scores greater than 0. The last step was implemented to emphasize local network activity. RESULTS/ANTICIPATED RESULTS: We report the results of a pilot study to test the performance of a new operator independent method for passive identification of CS with intractable epilepsy undergoing icEEG monitoring at Yale, in whom CS localization was obtained by standard methods. The sensori-motor (SM) cortex exhibited higher EEG-gamma power compared with non-SM cortex (*p*<0.0002). There was no significant difference between the motor/premotor and sensory cortex (*p*<0.47). CS was successfully localized in all patients with thresholds between 0.4 and 0.6. In 2 patients, knowledge of anatomy was needed to distinguish the MC from adjacent epileptic foci. The primary hand and leg motor areas exhibited the highest metric values consistently followed by the tongue motor area. Higher threshold values were very specific (94%) for the anterior bank of the CS but not sensitive. Intermediate threshold values achieved a reasonable trade-off (0.4: 89% specific and 70% sensitive). DISCUSSION/SIGNIFICANCE OF IMPACT: We present and successfully implement a rapid procedure for task-free and stimulation free localization of the central sulcus during sleep based on intrinsic electrophysiological properties of the primary motor strip which exhibits increased power and enhanced local connectivity. We successfully localized the central sulcus in all patients. When implementing appropriate thresholds, our proposed metric M is very specific for the anterior lip of the central sulcus which may make it ideal to identify this important anatomical landmark. Our method is sensitive for epileptogenic regions as well, therefore basic knowledge about central sulcus anatomy may be needed in cases where there is an epileptogenic lesion in the vicinity of the central suclus. Our method makes a few a priori assumptions: The regions around the central sulcus are adequately sampled and the occipital or parieto-occipital regions are not included in the analysis. In order for the method to function properly, nonsensori-MC should be sampled adequately as well. In the future, normative data could be generated for the composite product of connectivity×power which may replace within-patient *z*-scoring. Our method is rapid and can be implemented on short segments of ECoG data. The proposed method may be potentially used for identification of seeds in the motor cortex for subsequent network analysis and further studies may delineate its potential use in the operating room.

